# Basic Characteristics of Dose Distributions of Photons Beam for Radiotherapeutic Applications Using YAG:Ce Crystal Detectors

**DOI:** 10.3390/ma15217861

**Published:** 2022-11-07

**Authors:** Janusz Winiecki, Sandra Witkiewicz-Lukaszek, Paulina Michalska, Seweryn Jakubowski, Sergiy Nizhankovskiy, Yuriy Zorenko

**Affiliations:** 1 Medical Physics Department, Prof. Franciszek Łukaszczyk Oncology Center, Dr Izabeli Romanowskiej Street 2, 85-796 Bydgoszcz, Poland; 2 Department of Oncology and Brachytherapy, Collegium Medicum in Bydgoszcz Nicholas Copernicus University in Toruń, Jagiellońska Street 13/15, 85-067 Bydgoszcz, Poland; 3 Institute of Physics, Kazimierz Wielki University in Bydgoszcz, Powstańców Wielkopolskich Street 2, 85-090 Bydgoszcz, Poland; 4 Institute for Single Crystals, National Academy of Sciences of Ukraine, Avenue Nauki 60, 61178 Kharkiv, Ukraine

**Keywords:** thermoluminescence, YAG:Ce and LuAG:Ce crystals, X ray, radiotherapy

## Abstract

Thermostimulated luminescence (TSL) dosimetry is a versatile tool for the assessment of dose from ionizing radiation. In this work, the Ce^3+^ doped Y_3_Al_5_O_12_ garnet (YAG:Ce) with a density ρ = 4.56 g/cm^3^ and effective atomic number Z_eff_ = 35 emerged as a prospective TSL material in radiotherapy applications due to its excellent radiation stability, uniformity of structural and optical properties, high yield of TSL, and good position of main glow peak around 290–300 °C. Namely, the set of TSL detectors produced from the YAG:Ce single crystal is used for identification of the uniformity of dose and energy spectra of X-ray radiation generated by the clinical accelerator with 6 MV and 15 MV beams located in Radiotherapy Department at the Oncology Center in Bydgoszcz, Poland. We have found that the YAG:Ce crystal detects shows very promising results for registration of X-ray radiation generated by the accelerator with 6 MV beam. The next step in the research is connected with application of TSL detectors based on the crystals of much heavier garnets than YAG. It is estimated that the LuAG:Ce garnet crystals with high density ρ = 6.0 g/cm^3^ and Z_eff_ = 62 can be used to evaluate the X-rays produced by the accelerator with the 15 MV beam.

## 1. Introduction

The primary goal of radiotherapy is to deliver to the tumor tissue a dose of ionizing radiation that, in the classical sense of this method of treatment, will lead to apoptosis of cancer cells. Normal cells, even if spread within the tumor, usually survive the conventional treatment, as their mechanisms of self-repair are much more developed and effective than those of cancer cells.

The dose distribution within the selected part of patient’s body should correspond with the shape of tumor body (target). The healthy, surrounding organs (organs-at-risk (OAR)) should be consequently protected (e.g., by shields) to minimize the toxicity of the therapy. This approach in known as conformality.

Currently, very sophisticated methods are used to personalize the treatment (e.g., intensity modulated radiation therapy (IMRT) and volumetric modulated arc therapy (VMAT)). However, in the same way as in previous years, the treatment planning and personalization of the dose distribution requires knowledge of the quality of the therapeutic beam.

The two dosimetric parameters describing a therapeutic beam of radiation generated by a conventional medical accelerator are *uniformity* and *flatness.* These factors characterize the intensity (dose) of radiation in the plane perpendicular to the beam axis (CAX) [1,2,3,4,5,6].

Dose measurements are most often performed in water phantoms, the physicochemical properties of which are similar to those of soft tissue. For radiotherapy, an ideal dosimeter should have the following features: high accuracy, high precision, low detection limit, ability to detect radiation over an appropriate dose range, linear dose response, which should be independent of the dose rate and independent of the radiation energy and should allow the measurement of doses in a very small volume (high spatial resolution).

The thermostimulated luminescence (TSL) dosimetry is a versatile tool for the assessment of dose from ionizing radiation. For this purpose, various TL detectors can be used mainly based on LiF and Al_2_O_3_:C compounds. However, due to very high energies of accelerators beams (usually 6 MV and 15 MV of nominal accelerating potential (NAP)), the application of the conventional TSL detectors based on LiF or Al_2_O_3_:C is limited. Assuming that the X-ray absorption ability of the compound is proportional to ρ × Z_eff_^4^, such bottlenoses correspond to low density ρ and effective atomic numbers Z_eff_ of the noted TSL materials.

For this reason, TSL detectors in radiotherapy applications can be new TSL materials based on the crystals, crystal-film composites, and ceramics of well-known oxide materials with wide range of ρ × Z_eff_^4^ values. Such new materials for checking the uniformity of dose radiation in typical diagnostic and therapeutic procedures must be suitable for full absorption of radiation with different energies, starting from conventional X-ray sources with energy in the 5–80 KeV range up to 6 MV and 15 MV beams produced by the typical linac accelerators.

In our research, we will consider, first, the crystals, films, and composites of garnet compounds A_3_B_5_O_12_ (A = Y, Lu, Gd; B = Al, Ga), doped with different types of rare-earth dopants, which are widely used as laser media, scintillators, and cathodoluminescence screens. Due to the well-developed technologies of garnets production in the crystal, films, and composites forms, very high material uniformity can be obtained in the case of preparation of large sets of TSL detectors for diagnostic of the uniformity of therapeutic X-ray sources.

Single crystals of Ce^3+^ doped Y_3_Al_5_O_12_ (YAG) and Lu_3_Al_5_O_12_ (LuAG:Ce) garnets are currently considered for applications as fast and efficient scintillators due to their excellent radiation stability, high yield (~10–20 Ph/KeV), and small decay time (50–70 ns) [7,8,9,10,11]. The YAG:Ce and LuAG:Ce crystals are characterized by large (up to 0.1–25 at.%) content of Y_Al_ or Lu_Al_ antisite defects (ADs) and oxygen vacancies as a consequence of high-temperature (1970–2030 °C) growth of bulk crystals of these garnets from the melt in the inert (Ar) atmosphere. The Y_Al_ and Lu_Al_ ADs and charged oxygen vacancies in the YAG and LuAG crystals play the role of emission centers in the UV range and trapping centers as well [10].

Apart from the scintillation applications, YAG:Ce and LuAG:Ce crystals with ρ = 4.5 g/cm^3^; Z_eff_ = 35 and ρ = 6.75 g/cm^3^; Z_eff_ = 59, respectively, recently emerged as possible alternative materials for TL dosimetry due to high TSL signal and good position of main glow peak in the 250–300 K range after irradiation of different types of ionization radiation [7,12,13]. Moreover, the TSL and OSL properties of YAG:Ce and LuAG:Ce crystals were considered in detail [7,9,12,14,15]. Due to such properties, YAG:Ce and LuAG:Ce crystals can be a good candidates for radiative and chemical resistant detectors with small volume and high spatial resolution [16]. Furthermore, using the LPE growth method, the new types of composite TSL detectors based on the film–crystal epitaxial structures of YAG:Ce and LuAG:Ce garnets were recently developed for simultaneous registration of the different components of ionization radiation [7,13]. Namely, the separate detection of the different components of mixed ionization fluxes in such composite detectors occurs due to differences between the TSL glow curves after α- and β- particles or γ-rays excitation, recording from the film and substrate parts of YAG:Ce film/LuAG:Ce crystal and LuAG:Ce film/YAG:Ce crystal composite detectors [7].

In this work, we used the TSL properties of the YAG:Ce garnet crystals for clinical dosimetry applications with the aim to identify the uniformity of dose and energy spectra of X-ray radiation generated by the clinical accelerator with 6 MV and 15 MV beams located in the Radiotherapy Department at the Oncology Center (RD OC) in Bydgoszcz, Poland.

## 2. Samples and Equipment

For testing of a beam uniformity, we have used in this work three sets of YAG:Ce TSL detectors (each for four samples), prepared with the same Czochralski grown crystal. First, 12 samples of YAG:Ce crystals with the size 10 mm × 10 mm × 1 mm and relatively the same TSL properties were selected. The samples were irradiated with low (40–140 KV) and high (6 MV and 15 MV) energy X-rays as well as with 1.17 and 1.33 MeV high energy γ quanta from a ^60^Co source. For this purpose, an Acuity radiotherapy simulator (for low energy X-rays) and a Clinac 2300C/D linear accelerator (for high energy X-rays) both from VMS (Varian Medical System, Palo Alto, CA, USA) were used. The devices operate at RD OC in Bydgoszcz. The exposure to gamma radiation was carried out at the Department of Medical Physics, National Institute of Oncology in Warsaw (Theratron 780C from Best Theratronics Ltd., Kanata, ON, Canada).

**Figure 1:**
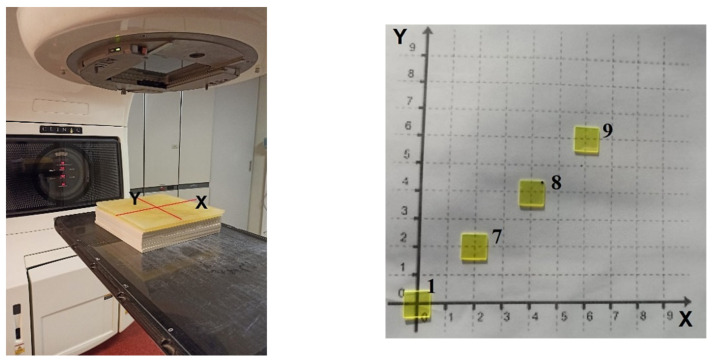
The position of the samples on the table of Clinac 2300CD Silhouette (Varian Medical Systems, Palo Alto, CA, USA) accelerator relative to the center of target (**left side**) and the position of the YAG:Ce detectors on the experimental x-y plane (**right side**). The positions of the detectors are also identified by numbers in the x-y plane of the right figure.

The arrangement of one set of YAG:Ce detectors (1, 7, 8, 9) is shown in Figure 1. The samples are positioned at 0 × 0; 2 × 2; 4 × 4; and 6 × 6 cm from the center of the axis (Figure 1).

**Figure 2:**
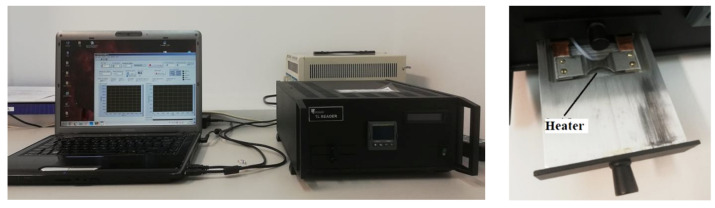
TSL reader at Chair for Optoelectronic Materials of the Institute of Physics Kazimierz Wielki University in Bydgoszcz.

The TSL glow curve measurements were performed using TSL-reader (production IP PAN, Warsaw, Poland) (Figure 2) under different ionizing radiation (see section “Ionizing Radiation”). The same procedures (the time from irradiation up to the measurement performance) were used during each cycle of measurement. The TL Reader software enables a heating rate of 5 degree/sec, and the final heating temperature was 400 °C. The detectors were always stored in the same conditions, protected from sunlight and other factors (temperature, humidity) that could affect the signal fading. The experimental error in the determination of the TSL glow peak position as well as the intensity of this peak was estimated and equal to 1.5–2 °C and 3–5%, respectively. 

A Schott BG 39 “green” filter was used in all measurements for separation of the Ce^3+^ luminescence in YAG:Ce detectors. The transmittance range of this filter, extending from 350 to 700 nm, was well matched with the emission range of the Ce^3+^ luminescence in the YAG:Ce crystals. Taking into account that Ce^3+^ ions typically serve as hole trapping centers in TSL processes in oxides, all the observed peaks in the TSL glow curves of YAG:Ce detectors are related to the electron trapping centers. Usually such centers in garnet crystals, grown from high temperature melts, are created by the antisite defects, oxygen vacancies, and their aggregates (see [9] for details).

## 3. Sources of Ionizing Radiation and Experimental Set-Up for Irradiation

**Figure 3:**
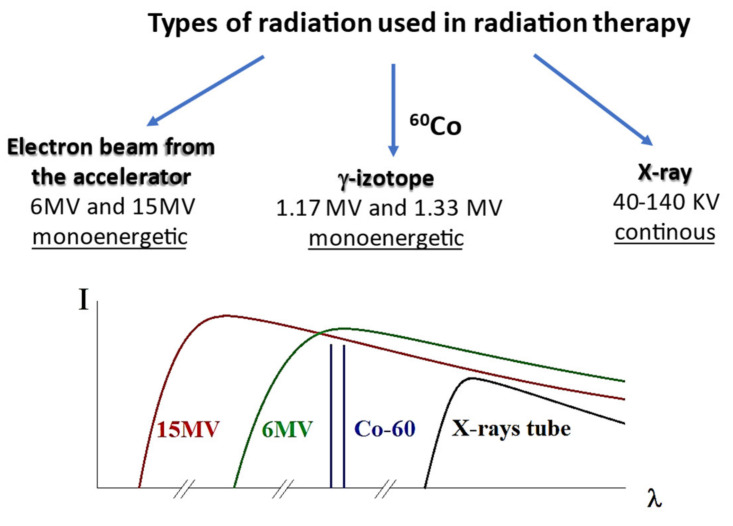
Different types of radiation used in this work (**top**); the schematic dependence of wavelength λ of X-rays on the type of radiation (**bottom**).

Generally, the forms of radiation relevant to the treatment of cancer are X- and g-rays as well as particle radiation beams (Figure 3). Those forms of radiation are either directly or indirectly ionizing the material of the target. Directly ionizing radiation (e.g., a beam of protons, alpha particles, or beta particles) causes direct disruption of the atomic or molecular structure of the tissue through which it passes. In contrast, indirectly ionizing radiation (e.g., electromagnetic waves and neutron beams) gives up energy as it passes through tissues, which results in the production of fast-moving particles that in turn causes damage to tissues.

Considering the above, three types of ionizing radiation were used in this work, namely (i) continuous low energy of X-ray sources working with the accelerating voltage in the 40–140 KV range and current in the 200–400 mA range; (ii) γ-rays with an energy of 1.17 and 1.33 MeV from the γ^60^Co isotope, and (iii) high-energy X-ray radiation generated by the clinical accelerator working with 6 MV and 15 MV accelerated electron beams (Figure 3).

The use of an electron beam for radiotherapeutic applications requires that the spatial distribution of the delivered dose (profile) be uniform in the irradiated volume and as small as possible outside of it. It follows, therefore, that an ideal decrease in the dose radiation should be stepwise at the boundary of the therapeutic field. Therefore, due to radiation scattering, the ideal rectangular shaped dose distribution cannot be obtained.

**Figure 4:**
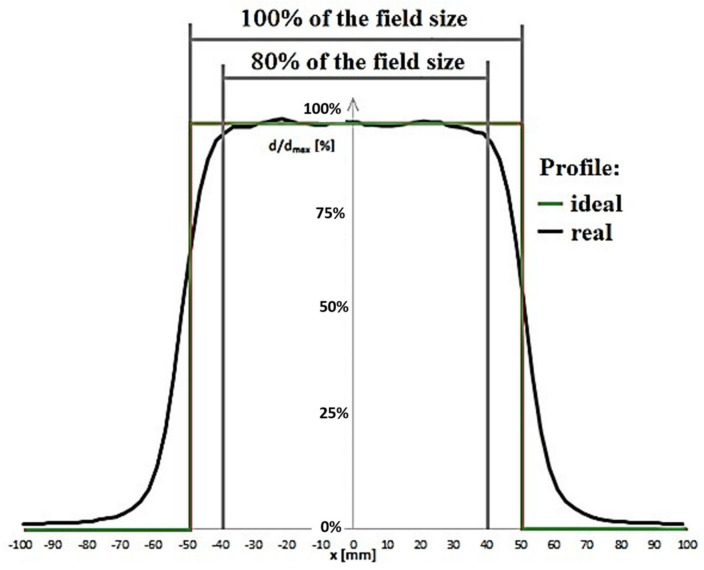
Typical beam profile for radiotherapeutic applications.

A parameter referred to as *flatness* is therefore defined. It is defined in water in a plane perpendicular to the axis beam, in the area of 80% of the dimensions of the irradiated field at a depth corresponding to the depth deposition of the maximum dose in the therapeutic beam axis (Figure 4). The presented relationships are the basic physical quantities that must be measured in order to prepare the patient for radiotherapy. These physical quantities must be well known before exposure of the patient to radiation and thus there is the need for qualifying the radiation field. Dose calculations are performed by computer programs known as treatment planning systems.

**Figure 5:**
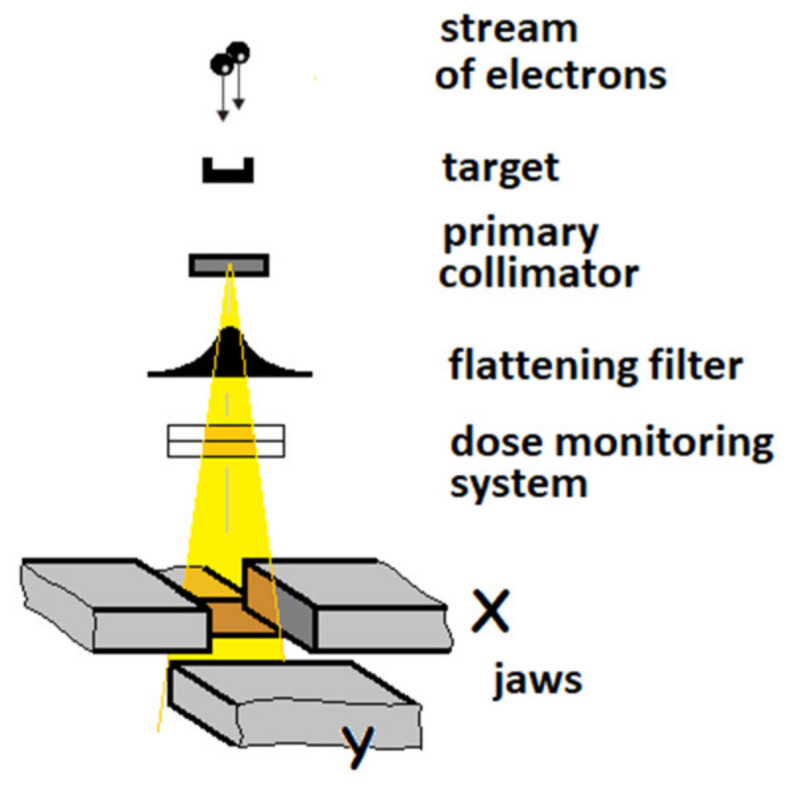
Diagram of the beam collimation system in a standard medical accelerator—Linac head.

Hard X-ray photons are produced by the conversion of the kinetic energy of the accelerated electrons in a tungsten target (***Bremsstrahlung***). The electrons produced by the electron gun and accelerated in a short, vertically positioned acceleration structure working with nominal acceleration potential (NAP) of 6 and 15 MV hit the target to produce the high energy X-ray beam. Subsequently, the X-ray beam passes from the pre-collimator and the flattening filter to obtain the so-called flat beam (Figure 5).

There are two ionization chambers below the filter that monitor the radiation dose. The initial and rotary collimator as well as its movable jaws allow for precise definition of the irradiation field. It is possible to attach an accessory shelf to the plate under the collimator, enabling additional shaping of the field. Namely, in our experiments, we have used the two types of 60°_Left and 60°_Right wedges from an alloy of iron with copper [17,18].

**Figure 6:**
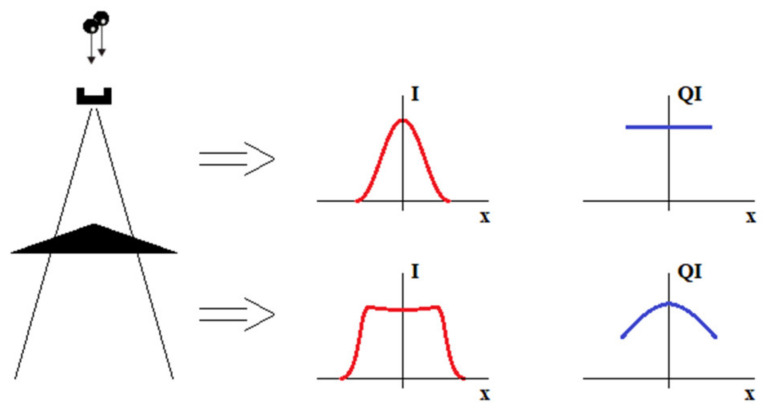
High energy X-ray (6 MV and 15 MV) intensity (I) and radiation quality (QI) off-axis characteristics at selected stages of therapeutic beam generation in a classic medical accelerator—simplification.

Figure 6 shows schematically the effect of a flattening filter in a classic accelerator. The newly formed X-ray beam has an approximately Gaussian *intensity distribution* (I) and approximately the same quality throughout the plane. As the therapeutic beam is a superposition of X-rays with different wavelengths (energies), the term “radiation quality” is related the beam’s spectral composition. In the clinical environment, the *Quality Index* (QI) parameter is used to describe the quality (energy) of radiation. The QI value is determined directly from the dependence of the absorbed dose (D) measured in the water at depth. The QI is a function of D_20_/D_10_, where D_20_ and D_10_ are the doses measured at a depth of 20 cm and 10 cm, respectively.

**Figure 7:**
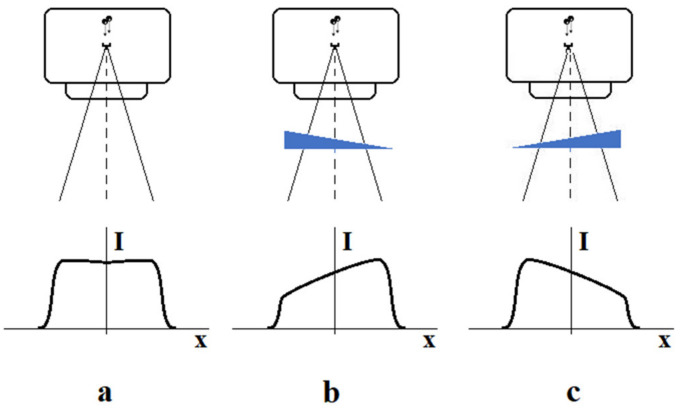
Influence of wedge filter on beam intensity distribution: (**a**) open field (no wedge), (**b**) left side wedge orientation, (**c**) right side wedge orientation.

The flattening filter makes the profile nearly flat, which is useful for simplifying the dose calculations. At the same time, some changes are observed in the radiation quality. Filtration makes the radiation at the beam axis harder than that at the periphery. In some clinical situations, a modification (skew) of the beam profile is required. This action aims to better irradiate the tumor or protect organs at risk. This is achieved by using hard wedges—consider Figure 7.

**Figure 8:**
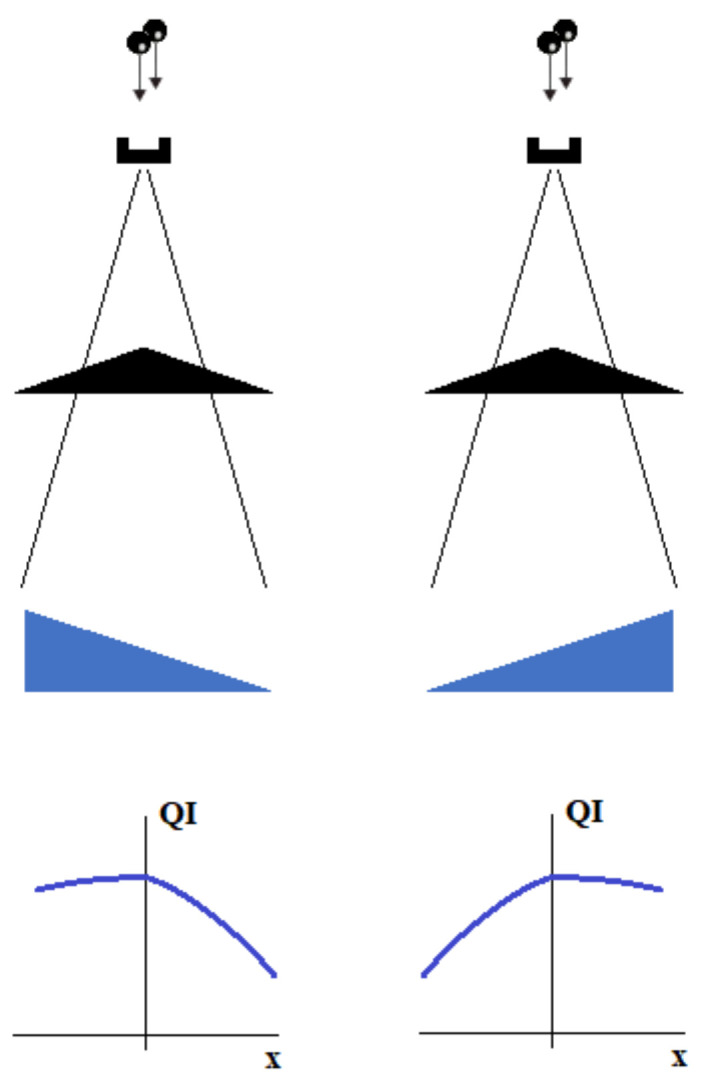
Effect of simultaneous work of flattening filter and hard wedge for both wedge orientations.

The use of an appropriate wedge may intensify or weaken the filter effect. As shown in Figure 8, the effective radiation quality factor may remain nearly constant on one side of the beam axis and rapidly drops down on the other. The efficiency of this effect will depend on the density of the wedge material and its inclination.

The scope of the experiment was to verify whether the samples used in the experiment would give a different response with varying radiation quality. For this purpose, the samples were irradiated with an open beam, without a wedge, and then with wedge at two opposite 60° orientations. It should be noted here that during the experiment, when the response to radiation of various qualities was tested, the dose received by individual samples was the same (2 Gy) despite the use of a wedge. This effect was obtained through a sufficiently long exposure time. Although all samples are shown together in Figure 1, each dose was delivered separately, which allowed for individual exposure time for the selected sample. The accuracy of determination of the delivered 2 Gy dose was 0.3%.

By ensuring that the same dose is delivered to each of the irradiated samples, we created conditions for testing the impact of the radiation quality on the detectors’ work. We assumed that changing the wedge orientation to the opposite would have a significant impact on the TSL signal readout, as the wedge would intensify the flattening filter effect in one position and reduce it in the opposite one.

## 4. Experimental Results

### 4.1. Irradiation by the Continuous X-ray Sources

**Figure 9:**
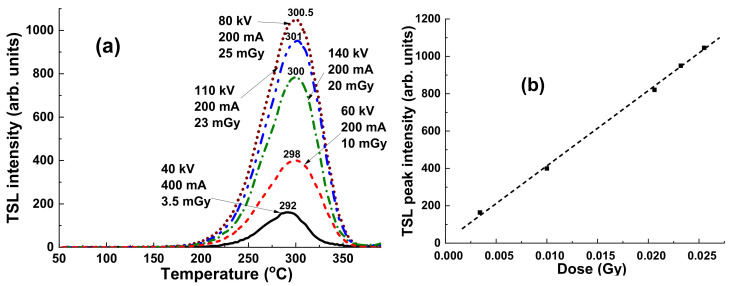
(**a**) Dependence of TSL peak intensity and peak position for YAG:Ce crystal sample on irradiation dose by continuous X-ray photons working with NAP in the 40–140 KV range and current in the 200–400 mA range; (**b**) TSL intensity as a function of the dose of irradiation for the YAG:Ce crystal detector.

Figure 9 presents the TL glow curves for a YAG:Ce crystal sample, previously irradiated with a dose in the 0.0034–0.0255 Gy range in the open field mode by a conventional X-ray source, working with variable acceleration potential (AP) in the 40–140 KV range and current in the 200–400 mA range. Despite the relatively low dose of irradiation, the intensity of TL is very high. All TSL curves show quite asymmetric shape but close peak positions in the 292–301 °C range (Figure 9a). The asymmetric shape of the main TL peaks is probably caused by the overlapping of two peaks around 220 K and 300 K (see Figure 13 for details). However, due to high overall intensity of TSL even at such a low dose of radiation, the low-temperature peak is strongly overlapping with the high-temperature one.

Of most importance here is the practically linear dependence of the main TL peak intensity on the dose of irradiation in the noted range. This means that the YAG:Ce crystals can be used as a suitable dosimeter for estimation of the beam uniformity at typical doses of radiation in radiology.

### 4.2. Results for 6 MV Irradiation

**Figure 10:**
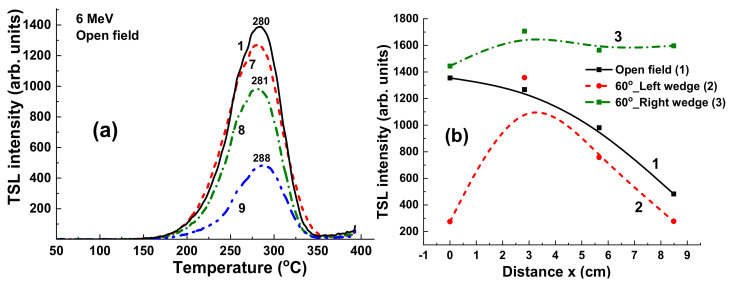
(**a**) TSL glow curves for YAG:Ce crystal samples irradiated by 6 MV X-ray photons from a Clinac 2300 accelerator in open field mode; (**b**) dependence of intensity of TSL peaks on the distance x between sample and centers of target (see Figure 1).

Figure 10 presents the TL glow curves for the set of YAG:Ce crystal samples, previously irradiated by 6 MV X-ray photons from a Clinac 2300 accelerator with a dose of 2 Gy in the open field mode without using wedges. As seen in Figure 10a, the TSL glow curves show similar shape and close peak positions in the 280–288 °C range. However, the main variations concern the TSL peak intensities due to differences in the energy of the X-ray radiation in different points of the target. Namely, a higher intensity is manifested in sample 1 in the center of target, whereas the smallest intensity is in sample 9 with the largest distance to the center of the target (Figure 10b, curve 1).

**Figure 11:**
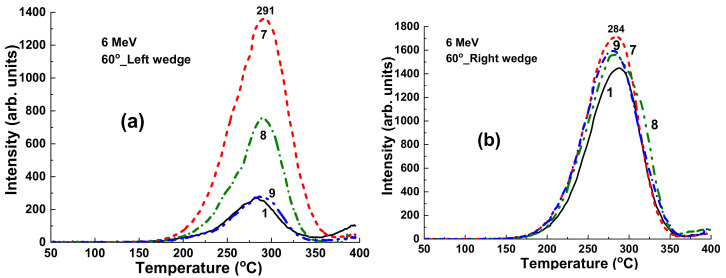
(**a**) TSL glow curves for YAG:Ce crystal samples irradiated by 6 MV X-ray photons from Clinac 2300 accelerator in the case of using additional 60°_Left (**a**) and 60°_Right (**b**) wedges (see Figure 8).

Using additional wedges with different shapes can strongly influence the radiation dose uniformity and intensify or weaken the flattening filter effect (Figure 11). Namely, application of a 60°_Left wedge significantly increases the differences in the intensity of the TSL glow peaks (Figure 11a). In contrast, using a 60°_Right wedge strongly decreases the deviation in the TL intensity of the samples in the different target positions (Figure 11b). The respective dependences of the TSL intensity of YAG:Ce crystals on distance between position of samples and the center of target x in the case of using 60°_Left and 60°_Right wedges are shown in Figure 11b, curves 2 and 3, respectively.

The results presented in Figure 10 and Figure 11 mean that the shape and intensity of the TSL glow curves are strongly influenced not only by the dose but also by the energy of radiation.

### 4.3. Results for ^60^Co Irradiation

**Figure 12:**
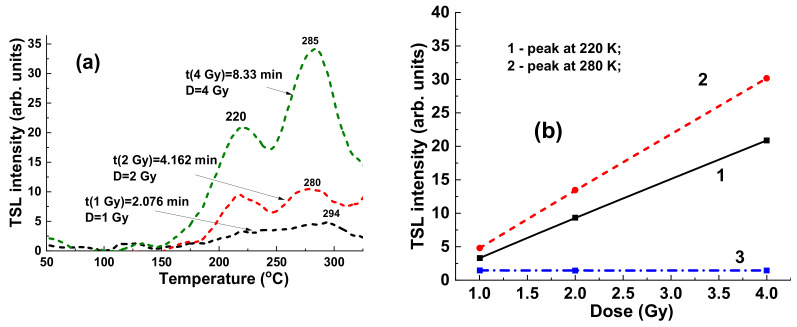
(**a**) Dependence of intensity of TSL glow curves for YAG:Ce crystal detector on the dose of irradiation by ^60^Co source; (**b**) intensity of TSL glow peaks of YAG:Ce crystal at 220 °C (1) and 285 °C (2) source and I_285_/I_220_ ratio of intensity of these peaks (3) on dose of γ rays irradiation by ^60^Co.

Figure 12 presents the TSL glow curves for YAG:Ce crystal samples irradiated by γ-rays with an energy of 1.17 and 1.33 MeV from ^60^Co sources with dose in the 1–4 Gy range in the open field mode. It is important to note here that the flattening filter is absent in the case of ^60^Co irradiation as opposed to the case of X-ray photon irradiation (Figure 9). All the TSL curves after ^60^Co irradiation (Figure 12a) show the well distinguished structure consisting of two peaks at 200 °C and in the 280–294 °C range. Interestingly, the intensity of low- and high-temperature TSL glow peaks are linearly dependent on the dose of radiation in the 1–4 Gy range (Figure 12b, curves 1 and 2, respectively) with quite different slopes. At the same time, the ratio between the intensity 285 °C/220 °C peaks being equal to 1.3 is constant with respect to the dose of irradiation. Furthermore, the intensity of TSL of YAG:Ce crystal samples is substantially less in the case of ^60^Co irradiation in the open field mode (Figure 12a) than that for 6 MV radiation (Figure 9a), probably due to the lack of the low-energy part of the X-ray spectra for 6 MV linac beam.

### 4.4. Results for 15 MV Irradiation

**Figure 13:**
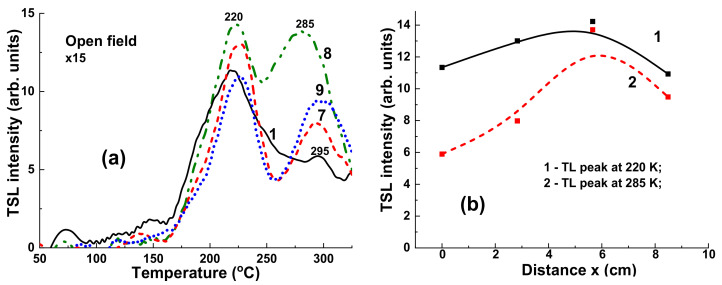
(**a**) TSL glow curves for YAG:Ce crystal detector irradiated by 15 MV X-ray photons from a Clinac 2300 accelerator in open field mode; (**b**) dependence of the intensity of two TSL peaks of YAG:Ce crystal at 220 °C (1) and 285 °C (2) on the distance x between sample and centers of target.

Under irradiation by 15 MV X-rays in the open field mode, the low and high temperature peaks at 200 °C and in the 280–294 °C range show quite similar dependences on the place of localization of the YAG:Ce samples onto the target (Figure 13a). However, the most important characteristic here is the sharp and non-linear character of such dependences (Figure 13b). Such results confirm the aforementioned conclusion about limitations in the application of YAG:Ce crystal detectors with medium density and effective atomic number in the case of high energy (15 MV) X-ray excitation.

**Figure 14:**
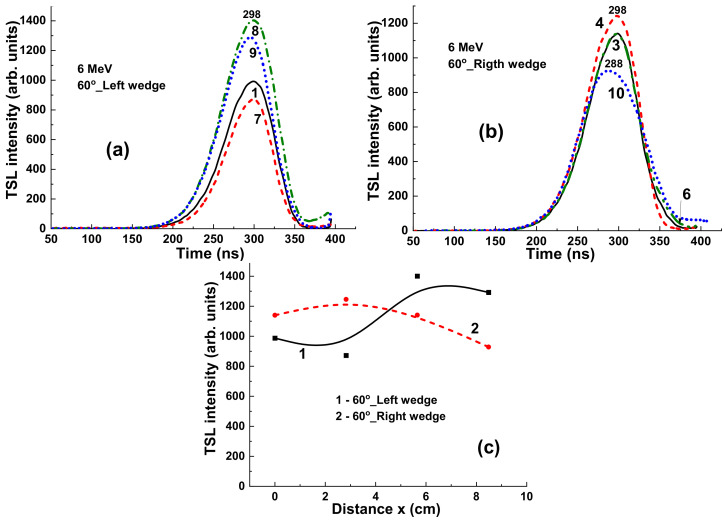
(**a**,**b**) TSL glow curves for YAG:Ce crystal detectors irradiated by 15 MV X-ray photons from a Clinac 2300 accelerator in the case of using additional wedges 60o_Left (**a**) and 60o_R-right (**b**); (**c**) dependence of the intensity of TSL peaks on distance x between sample and center of target (see Figure 1 and Figure 9).

However, when the high energy part of the 15 MV X-ray irradiation is absorbed by the 60°_Left and 60°_Right wedges (Figure 14a,b, respectively), the TSL intensity of YAG:Ce detectors strongly increases, and such crystals can be used adequately for measurements of the uniformity of the beam. Namely, using 60°_Left wedge, the TSL intensity of detectors decreases with the distance x between the sample and centers of target; whereas in the case of 60°_Right wedge application, the TSL intensity of detectors increases with distance of sample localization (Figure 14c, curves 1 and 2, respectively).

**Figure 15:**
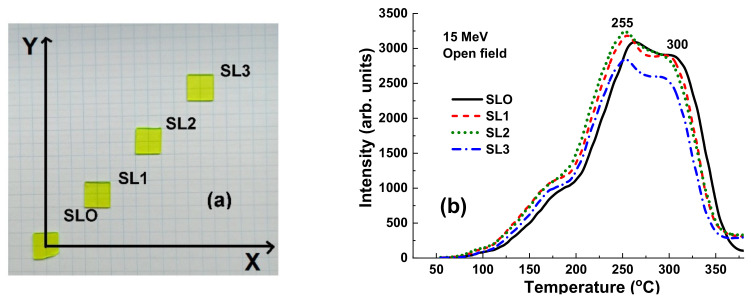
(**a**) Position of LuAG:Ce crystal samples on the experimental x-y plane. (**b**) TSL glow curves for set of LuAG:Ce crystal detectors irradiated by 15 MV X-ray photons from a Clinac 2300 accelerator in the open field mode.

The aforementioned results with γ-rays irradiation by ^60^Co source (Figure 12) and 15 MV X-ray irradiation (Figure 13) mean that YAG:Ce crystals are not so convenient materials for investigation of beam uniformity at therapeutic radiation treatment by X-rays with high energies (above 6 MV) in the open field mode and can be substituted or combined with a heavy analogue of such material as LuAG:Ce garnet with ρ = 6.73 g/cm^3^ and Z_eff_ = 62 [2]. Currently, such research is in progress, and preliminary results are very encouraging (Figure 15). The LuAG:Ce crystal detectors show the high TSL intensity in the open field mode with small deviation in the intensity of the main peaks at 255–300 K on the position of the staples with respect to the center of target.

Furthermore, following the main results of our previous work [2], the creation of a universal TSL composite detectors based on the LPE grown YAG:Ce film/LuAG:Ce crystal epitaxial structures is considered as well for estimation of beam uniformity obtained by accelerators working with both 6 MV and 15 MV NAP.

## 5. Conclusions

The TSL properties of the YAG:Ce garnet crystals were used in this work for radiotherapy applications with the aim of identifying the uniformity of dose and energy spectra of X-ray radiation generated by clinical radiotherapeutic beams working with nominal accelerator potential (NAP) of 40–140 KV, 6 MV, and 15 MV and γ-rays produced by ^60^Co source with an energy of 1.17 and 1.33 MeV. The YAG:Ce crystal detector shows the high TSL light yield and linear dependence of the intensity of the main TSL peak at 290–300 °C on the dose of X-ray irradiation in the 3–25 mGy range, produced by the continuous X-ray source with NAP in the 40–160 kV range, working in the open field mode. The intensity of the main TSL peaks of YAG:Ce crystals in the 200–300 °C range shows good linearity with the time and dose of higher energy γ-ray radiation produced by the ^60^Co source. We also found that the set of TSL detectors, performed with the same YAG:Ce crystal, can be used as suitable dosimeters for estimation of a beam uniformity in the 6 × 6 cm^2^ target field at typical doses of therapeutic radiation treatment of 2 Gy produced by the clinical accelerator working with NAP of 6 MV.

However, the intensity of TSL of YAG:Ce crystals is substantially less in the case of registration of γ-rays produced by a ^60^Co source with an energy of 1.17 and 1.33 MeV and X-ray radiation generated by accelerator with higher nominal NAP of 15 MV in the open field mode than that in case of 6 MV radiation due to relatively low density ρ = 4.5 g/cm^3^ and effective atomic number Z_eff_ = 35 of the detector material. This means that, for investigation of beam uniformity at higher energies (15 MV) of therapeutic radiation treatment, the YAG:Ce crystal detectors must be substituted or combined with heavier analogues of such TSL material. Such materials can be crystal LuAG:Ce garnet with ρ = 6.73 g/cm^3^ and Z_eff_ = 62. The preliminary results of testing of these TSL materials with a 15 MV therapeutic beam are encouraging and confirm our conclusion.

## Data Availability

Not applicable.
